# A simple protocol for producing axenic seeds of *Sorghum bicolor*

**DOI:** 10.17912/micropub.biology.001772

**Published:** 2025-09-09

**Authors:** Beatrice Bock, Jack Scherer, Faith Parrish, Julia Burnside, Callum Rohrer, Catherine Gehring

**Affiliations:** 1 Biological Sciences, Northern Arizona University, Flagstaff, Arizona, United States; 2 Center for Adaptable Western Landscapes, Northern Arizona University, Flagstaff, Arizona, United States

## Abstract

Microbes within seeds can confound research on microbial colonization, symbiosis, and pathogenesis. Sterilization of both external and internal seed tissues is therefore essential in certain experiments, but the method must also preserve seed viability. Here, we present a reliable and simple protocol for sterilizing *Sorghum bicolor* seeds by submerging them in 95% ethanol for 2 minutes followed by 3.75% sodium hypochlorite for 20 minutes. This approach yielded a low contamination rate (2 out of 95 seeds) and a robust median germination rate (63%). Its simplicity, cost-effectiveness, and accessibility make it a practical option for experiments requiring axenic seeds.

**Figure 1. Prevalence of germination and contamination among Sorghum bicolor seeds treated with the described sterilization protocol:**
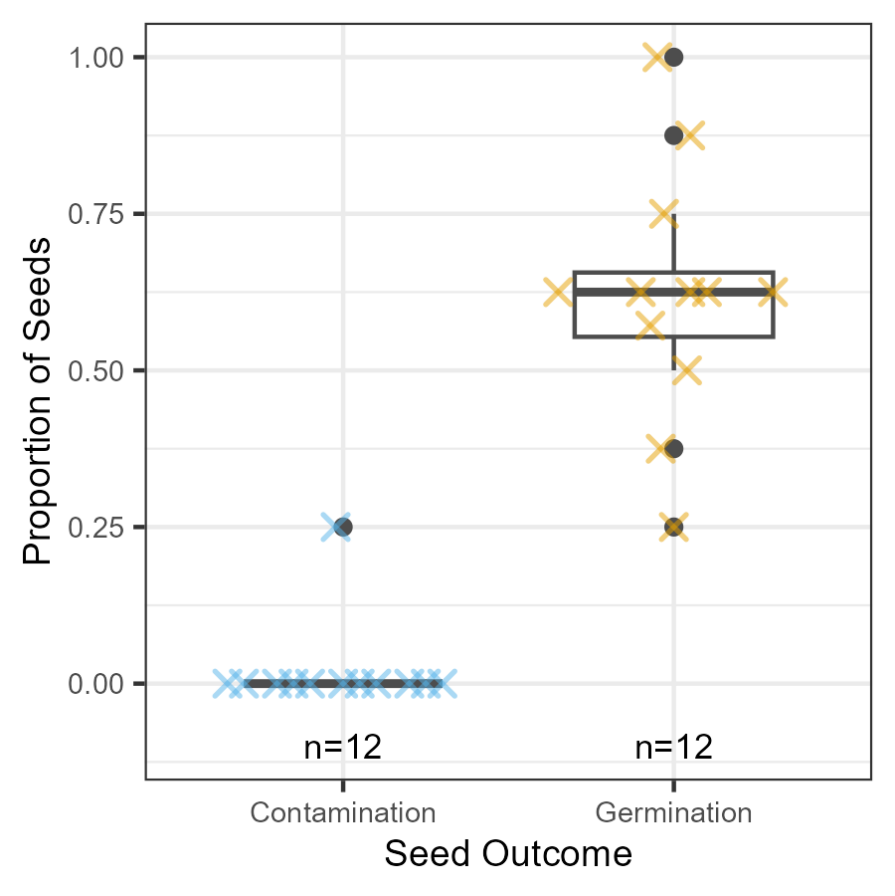
Each X symbol represents a group’s observed proportion of germinated or contaminated seeds. Each group consisted of 7-8 seeds treated at the same time with the described method. Of the 95 seeds tested, only two showed contamination by culturable microbes. Both seeds came from the same group. Boxplot elements include a horizontal line at the median (contamination = 0, germination = 0.63), a box showing the interquartile range (IQR), whiskers extending to values within 1.5 × IQR, and black circles marking outliers.

## Description

For studies involving microbial inoculation, colonization, or plant-microbe interactions, axenic seeds are helpful to avoid confounded results (e.g. Bock et al., 2025). Seed sterilization methods have been developed for many plant species but often fail to eliminate internal, culturable microbes (Abdelfattah et al., 2022; dos Reis et al., 2022). Thus, a method that reliably removes culturable microbes from both internal and external tissues without compromising germination is needed. Developing reliable and low-cost axenic seed protocols not only advances research in plant-microbe interactions, but also supports breeding programs, bioassays, and studies of seed-borne pathogens across diverse crop species.

We tested a simple protocol to produce axenic seeds of *Sorghum bicolor* . Groups of 7–8 seeds were placed in each of 12 test tubes. All subsequent steps were conducted in a sterile biosafety cabinet. Each group was shaken by hand in 95% ethanol for 2 minutes, then the ethanol was replaced with 3.75% sodium hypochlorite. This concentration is roughly equivalent to half-strength household bleach. Seeds were immersed in bleach for 20 minutes, a modification from Oyebanji et al. (2009), who exposed seeds to bleach for 30 minutes. Other exposure times were not tested, as this protocol was already in use in our laboratory; we shortened the duration to maximize germination while maintaining effective sterilization. After treatment, the bleach was poured off, and seeds were rinsed twice with autoclaved, sterile water. Seeds were then plated on Sigma Aldrich Phytagel™, a plant cell culture medium that can be used in place of agar and allows for easy detection of microbial contamination. To rule out external sources of contamination, plating was performed within a biosafety cabinet; gloves were cleaned with 70% ethanol, and all tools were dipped in 70% ethanol and flame sterilized between uses. Extra plates were also prepared without seeds, and no microbial growth was observed on them, confirming that contamination arose only from the seeds themselves or seed addition to the plates. In contaminated cases, microbial growth emerged spatially from the seed surface. Seeds were plated on 100 × 15 mm VWR Petri Dishes (polystyrene disposable sterile, slipable; Cat. No. 25384-302), with 7–8 seeds per plate. Seeds were spaced approximately 2–3 cm apart in a rough circular pattern. Plates were sealed with Parafilm® and incubated in a growth tent under 500 lumens of light (15 h light/9 h dark) at 22–32°C and ambient humidity (~30% relative humidity). Seeds were allowed to germinate on the plates for 7 days before they were used in a separate experiment, where they were inoculated with fungal isolates and grown for four weeks. No growth defects were observed during this period.

We selected *Sorghum bicolor* due to its global importance as a food and bioenergy crop (Calviño & Messing, 2012; Khalifa & Eltahir, 2023), climate resilience (Hossain et al., 2022), and documented seed endophytes (Kinge, 2019). Our protocol builds on that of Oyebanji et al., 2009, who used 90% ethanol for 3 minutes followed by 3.5% bleach for 30 minutes. However, their study did not report germination or post-germination contamination. By quantifying both, our study provides critical improvements for axenic seed protocols.

We processed 95 seeds in total, working in small groups of students. Contamination and germination were assessed 7 days after plating the seeds. Contamination, defined as visible microbial growth emanating from the seed, was rare: only 2 seeds out of 95 showed microbial growth, and both came from a single student group, suggesting a possible handling error rather than a flaw in the protocol. The contamination was not identified to type, though it visibly originated from the seed. All other seeds showed no microbial growth even after germination, indicating that the method was effective at eliminating culturable microbes. Germination was also high, with a median germination rate of 63%, which is sub-optimal for sorghum seeds but reasonable considering the exposure to ethanol and sodium hypochlorite (Sari & Juniarti, 2023).

This protocol reliably eliminates culturable microbes from *Sorghum bicolor* seeds while preserving viability. It is fast, low-cost, and does not require specialized equipment like sonicators (Burgdorf et al., 2014). While untested in other species, it may be broadly adaptable. Importantly, this method eliminates living, culturable microbes but does not necessarily remove microbial DNA or non-culturable taxa, which are considerations for molecular studies. For experiments concerned with active colonizers or microbial interactions, this protocol offers a practical and effective solution.

## Reagents

Seed source: 9300 Grain Sorghum Seed from Hancock Seed Co (hancockseed.com/products/9300-grain-sorghum-seed)

3.75% Sodium hypochlorite

95% Ethanol

Sigma Aldrich Phytagel™

100 × 15 mm VWR Petri Dishes (polystyrene disposable sterile, slipable; Cat. No. 25384-302)

Parafilm® M Sealing Film Roll
